# Intervention Characteristics that Facilitate Return to Work After Sickness Absence: A Systematic Literature Review

**DOI:** 10.1007/s10926-012-9359-z

**Published:** 2012-04-04

**Authors:** Nicole Hoefsmit, Inge Houkes, Frans J. N. Nijhuis

**Affiliations:** 1 Department of Social Medicine, Researchschool Caphri, Maastricht University, P.O. Box 616, 6200 MD Maastricht, The Netherlands; 2 Department of Work and Organizational Psychology, Maastricht University, P.O. Box 616, 6200 MD Maastricht, The Netherlands

**Keywords:** Intervention, Return to work, Sick leave, Absenteeism, Systematic review

## Abstract

*Introduction* In many Western countries, a vast amount of interventions exist that aim to facilitate return to work (RTW) after sickness absence. These interventions are usually focused on specific target populations such as employees with low back pain, stress-related complaints or adjustment disorders. The aim of the present study is to detect and identify characteristics of RTW interventions that generally facilitate return to work (i.e. in multiple target populations and across interventions). This type of knowledge is highly relevant to policy makers and health practitioners who want to deliver evidence based care that supports the employee’s health and participation in labour. *Methods* We performed a keyword search (systematic literature review) in seven databases (period: 1994–2010). In total, 23 articles were included and assessed for their methodological quality. The characteristics of the interventions were evaluated as well. *Results* Early interventions, initiated in the first 6 weeks of the RTW process were scarce. These were effective to support RTW though. Multidisciplinary interventions appeared effective to support RTW in multiple target groups (e.g. back pain and adjustment disorders). Time contingent interventions in which activities followed a pre-defined schedule were effective in all physical complaints studied in this review. Activating interventions such as gradual RTW were effective in physical complaints. They have not been studied for people with psychological complaints. *Conclusions* Early- and multidisciplinary intervention and time-contingent-, activating interventions appear most effective to support RTW.

## Introduction

Work can be beneficial for people’s health, reversing the harmful effects of prolonged sickness absence on the employee’s well-being. Improving the health and well-being of the working age population is critically important for individuals, organizations and society as a whole, in order to secure both higher economic growth and increased social justice [1]. In many Western countries, a large number of interventions exists to facilitate and hasten return to work (RTW) after sickness absence. These interventions include for example cognitive behavioural therapy [2], graded activity [3] and workplace adaptations [4].

Until now, systematic literature reviews that examined which interventions improved RTW, often focused on one diagnosis such as people with low back pain [5] or one intervention type such as interventions initiated by or integrated into the workplace, such as ergonomic work site visits [6]. However, we do not know yet whether and which intervention characteristics are generally effective, and therefore can be included in RTW interventions for multiple target populations. Therefore, the aim of this study is to detect and identify characteristics of RTW interventions that generally facilitate return to work (i.e. in multiple target populations and across interventions). Effective characteristics are part of RTW interventions that facilitate and hasten RTW, and at the same time are absent in interventions that do not facilitate RTW. We define facilitated RTW as either a significant reduction in the cumulate or mean number of (work, calendar or annual) days or weeks of sickness absence (whether or not measured at a certain follow up date) or an increase in work resumption rates (percentage of participants who resumed work partially or fully at a certain follow up date within the study period).

A problem, however, in this study is that standards by which we can classify RTW interventions do not exist yet. Therefore, we developed our own list of characteristics by which we classify the modern interventions that have been developed over the past two decades. This classification is based on earlier research [2, 4, 5] and consultations with other expert researchers. It appeared that modern RTW interventions can be characterized by one or more of the following characteristics:

- Timing of intervention: early, initiated in the first 6 weeks of absence or not;
- Care professionals involved: multidisciplinary, including multiple professionals (care providers) from more than one discipline or not;
- Planning of activities to support RTW: time contingent, in which activities are performed according to a pre-defined schedule or not;
- Target population: all employees on sickness absence irrespective of their specific medical diagnosis (generic) or only to employees with a specific diagnosis (specific);
- Character of activities to support RTW: interventions including explicit actions to stimulate the employee to RTW, which are A: whether or not a decision was made as to when and/or how RTW will take place; B: whether or not there was gradual exposure to the workplace; and C: whether or not workplace adaptations were implemented;
- Intensity: a high (≥10 h divided over multiple sessions), moderate (<10 h divided over multiple sessions) or low intensity (once);
- Employee and employer role: decision latitude of the employee and/or employer about activities to support medical recovery or RTW and the timing of RTW or no decision latitude of the employee and/or employer.

Knowledge about intervention characteristics that facilitate RTW is highly relevant to the sick and absent employee who wants to consume care that optimally improves his/her health and the employer who aims to reduce productivity losses. Moreover, health, social security and insurance policy makers and practitioners can use this knowledge to deliver evidence based care that supports the employee’s health and labour participation, thereby preventing future care consumption and dependence on benefits.

## Methods

### Search

We performed a systematic literature review. First, we searched Pubmed using the MeSH terms ‘absenteeism’, ‘sick leave’, ‘absenteeism AND intervention studies’, ‘sick leave AND intervention studies’. We restricted the first two searches to studies in which the search terms were a ‘major topic’. We searched for articles covering our keywords somewhere in the title, abstract or text body. Table 1 shows the results of this search. Because searching Pubmed using the MeSH terms yielded only 6 relevant studies, we performed a broader keyword search in Pubmed, CINAHL, PsycINFO, Cochrane Library and Google Scholar. We searched all these databases by using various combinations of the following keywords: ‘return to work’, ‘sickness absence’, ‘early’, ‘intervention’, ‘occupational’, ‘work’, ‘training’, ‘low’, ‘back’, ‘pain’, ‘whiplash’, ‘resumption’, ‘disability management’, ‘ergonomic’. Table 1 only shows only those keyword searches that yielded positive results. Titles and/or abstracts were screened until saturation (200 irrelevant hits in a row) was reached. We again searched for articles covering our keywords in the title, abstract or text body. We restricted the Cochrane search to reviews and the Google Scholar Search to the subject areas of Social Sciences, Arts and Humanities.

**Table 1:** Databases, search terms, hits and included publications

Database	Key words	Number of hits	Number included
Pubmed (MeSH)	Sick leave	310	6
	Sick leave AND intervention studies	8	0
	Absenteeism	214	0
	Absenteeism AND intervention studies	7	0
Pubmed	Return to work	4560	7
	Sickness absence	1065	2
Cinahl	Return to work	248	1
Cochrane library	Return to work	63	1
Google scholar	Return to work	About 625.000	3
	Return AND to AND work AND intervention	About 111.000	2
	Early AND return AND to AND work	About 190.000	1
Total			23

This procedure covered mostly recent articles, given the fact that the databases presented these first. Studies were included when they:

- Covered the effectiveness of interventions on RTW;
- Described interventions tested in a population of workers on sickness absence;
- Were full text articles;
- Were written in English and published in the last 16 years (from 1994 to 2010);
- Were empirical studies or systematic literature reviews.

We included systematic literature reviews to enlarge the body of evidence covered by this study. Such a large body of evidence is needed considering the broad scope of our study subject: to identify intervention characteristics facilitating RTW in multiple target populations (e.g. the employee on sickness absence with low back pain, psychological complaints, physical complaints etcetera). In total, 23 studies (18 quantitative studies and 5 systematic reviews) were included in this review.

We screened all literature lists of systematic literature reviews for overlap with the included empirical studies. In total 2 systematic reviews did not have any overlap with other empirical studies and 3 other reviews showed 3, 6 and 7% overlap with empirical studies. Considering these relatively small percentages, we included both the systematic reviews and the empirical studies. We also searched the literature lists of the systematic reviews for other relevant articles that met the inclusion criteria. This search resulted in the inclusion of one additional empirical study [7].

### Analyses

We assessed the methodological quality of all selected articles by means of the rating scheme presented in Table 2. Separate criteria were used for quantitative studies [largely based on 8] and systematic reviews [largely based on 9]. The criteria for quantitative studies are largely based on an existing tool from the Effective Public Health Practice Project [8]. The inter-rater reliability of the final grade assigned by this tool is considered excellent (intra-class correlation coefficient = 0.77, 95% Confidence Interval 0.51–0.90) [10]. We took the methodological quality of the articles into account in our description of effective interventions by attaching more value to the higher-quality studies. In case of inconsistent evidence, we attached more value to the high-quality studies.

**Table 2:** Criteria for evaluating the quality of empirical research

Research	Criteria		Evaluation									
Quantitative^a^	Design (A), measurements (B)	A	Randomized controlled trial (++)	Cluster randomized trial (++)	Controlled trial or quasi-experiment (+)	Longitudinal observational study (+)	Cross sectional study (−+)		Ex-post facto chart review (−+)		Case/case series study (−+)	
		B	>1 pre or post (++)	1 pre or post (+)	Multiple (+)	No (−+)						
	Study population	≥100 respondents (yes: ++, no: −+)					Heterogeneous: respondents of different ages, gender and/or educational levels (yes: ++, no: −+)					
	When applicable: control group	Description (yes: ++/no: −+)			Appropriate to draw conclusions: control group received nothing or care as usual differed from the intervention (yes: ++/no: −+)				Expected group difference described (yes: ++/no: −+)			
	Outcomes, measurement instruments	Outcomes described (yes: +/no: −+)	Outcomes match study aims (yes: +/no: −+)		Instruments described (yes: +/no: −+)		Validity and reliability of instruments described or logically no description (yes: ++/no: −+)		Instruments are likely to be accepted in the profession under concern (yes: +/no: −+)		References given for instruments (yes: +/no: −+)	
	Data analysis (scoring of most advanced method applied in study under concern)	Hierarchical regression with all variables measured at all moments (++)	Hierarchical regression without measurement of all variables at all moments (+)	Multiple regression analysis or multivariate analysis of variance (+)	Survival analysis (+)	Ex-post factor chart (+)	Descriptive statistics (−+)	Logistical regression (−+)	Regression analysis or analysis of variance (−+)	Correlation (−+)	Chi-square (−+)	T-test (−+)
Review^b^	Research question includes	Patient (yes: ++/no: −)			Intervention (yes: ++/no: −)			Comparison (yes: ++/no: −)			Outcome (yes: ++/no: −)	
	Search, selection	Sufficient terms (yes: ++/no: −)	Terms relevant for research question (yes: ++/no: −)	Search in databases as Medline (yes: ++/no: −)	Restrictions described (yes: ++/no: −)	Inclusion criteria described (yes: ++/no: −)	Inclusion criteria match research question (yes: ++/no: −+)	Selection by multiple researchers (yes: ++/no: −+)	Multiple researchers selected the articles independently (yes: ++/no: −+)		Selection process well described (yes: ++/no: −)	
	Quality rating	Quality rated (yes: ++/no: −)	Rating criteria described (yes: ++/no: −)		Multiple researchers (yes: ++/no: −+)		Multiple researchers rated the articles independently (yes: ++/no: −+)		Consensus seeking process between researchers described (yes: ++/no: −+)		Results reported for each included article separately (yes: ++/no: −)	
	Data extraction	Described (yes: ++/no: −)					Multiple researchers (yes: ++/no: −+)					
	Description original methods	Study population (yes: ++/no: −+)			Intervention (yes: ++/no: −+)		Outcomes (yes: ++/no: −+)		Confounders (yes: ++/no: −+)		Results (yes: ++/no: −+)	
	Description meta-analysis	Meta-analysis performed or heterogeneity described (yes: ++/no: −)					Potential sources of heterogeneity described (yes: ++/no: −)					

− = Minus one, insufficient; −+ = zero, neutral/sufficient; + = one, good; ++ = two, very good. A criterion is also ranked with a −+ in case it was inapplicable to the article or in case it cannot be identified based on the text in the article

^a^Effective Public Health Practice Project, n.d.

^b^Cochrane, n.d.

As regards the effectiveness of the interventions, data was extracted by reading and summarising articles. We used a standardised form that was developed for the purpose of this study. This form covered a description of the intervention and intervention characteristics, definitions of RTW/sickness absence and findings about the effectiveness of interventions. We included some systematic reviews in our study. We only read the primary studies in case the review article did not provide us all information needed to complete our form for data extraction.

To study the intervention characteristics that improve RTW, we defined several characteristics and developed a rating scheme by which we assessed all studies (Table 3). When descriptions of original studies were insufficient to rate a characteristic, we did not take this study into account in the results for this characteristic. For the systematic literature reviews, we rated whether the characteristics applied to one or more original studies included in those reviews. When this was the case, we took the results of these original studies into account in our results. In case a characteristic such as timing of the start of intervention varied largely across the original studies in the review, we rated this characteristic as neutral.

**Table 3:** Criteria for evaluating the characteristics of the included interventions

Intervention characteristic	Evaluation			
1. Timing of intervention, early which starts within first 6 weeks of absence	Yes	No or timing not restricted		
2. Care professionals involved, multidisciplinary, involving multiple professionals (care providers) from more than one discipline	Yes	No		
3. Planning of activities to support RTW, time-contingent, activities followed pre-defined time schedule	Yes	No		
4. Target population	Generic: all employees on sickness absence irrespective of their specific medical diagnosis	Specific: only employees with specific diagnosis		
5. Character of activities to support RTW, interventions including explicit actions to stimulate the employee to RTW	A: making decisions about actual RTW (when and/or how)	B: gradual exposure to the workplace (for example when employees resume work for a limited but increasing number of hours per week)	C: implemented work-related adaptations, e.g. workplace (such as ergonomic improvements of furniture)	
	Yes/no	Yes/no	Yes/no	
6. Intensity	High: ≥10 h divided over multiple sessions	Moderate: <10 h, multiple sessions	Low: once	Variable
7. Employee and employer role, decision latitude of the employee and/or employer about activities to support medical recovery or RTW and (the timing of) RTW	Yes	No		

Not described means that a certain characteristic is either not a part of the intervention or not described in the article. Not described is evaluated as a ‘no’

Systematic reviews were evaluated by reading the descriptions of original studies that were included in the reviews. A ‘no’ was also attached in case original intervention studies varied largely or in the case of doubt

The search and data analyses were discussed with peers. Please contact the corresponding author for more information about these procedures.

## Results

### Methodological Quality of the Studies

Table 4 shows the methodological quality of the studies included in this review.

**Table 4:** Methodological quality of the included studies

Quantitative study (in order of quality)	Design	Population	Control group	Outcomes, instruments	Data analysis	Score^a^
Brouwers et al. [11]	++	++	++	+	+	8 (Good)
van der Feldtz-Cornelis et al. [12]	++	++	++	+	+	8 (Good)
Mortelmans et al. [13]	++	++	++	+	+	8 (Good)
Bogefeldt et al. [18]	++	++	+	+	+	7 (Good)
Bültmann et al. [20]	++	++	++	+	−+	7 (Good)
Fleten and Johnsen [24]	++	++	+	+	+	7 (Good)
van der Klink et al. [16]	++	++	+	+	+	7 (Good)
Arnetz et al. [19]	++	++	+	+	−+	6 (Good)
Bakker et al. [29]	++	+	+	+	+	6 (Good)
Drews et al. [32]	+	++	+	+	+	6 (Good)
Hagen et al. [30]	++	++	+	+	−+	6 (Good)
Nystuen and Hagen [31]	++	++	+	+	−+	6 (Good)
Braathen et al. [25]	+	++	+	+	−+	5 (Moderate)
Marhold et al. [23]	++	−+	+	+	+	5 (Moderate)
Grossi and Santell [22]	++	−+	+	+	−+	4 (Moderate)
Godges et al. [26]	+	+	−+	+	−+	3 (Moderate)
Matheson and Brophy [21]	−+	++	−+	+	−+	3 (Moderate)
Weiler et al. [15]	+	−+	−+	+	+	3 (Moderate)

Review (in order of quality)	Research question	Search	Quality evaluation	Data extraction	Description methods original studies	Meta-analysis	Score^a^
van Oostrom et al. [14]	++	++	++	++	++	++	12 (Very good)
Carroll et al. [17]	++	++	+	++	++	++	11 (Very good)
Meijer et al. [7]	+	+	+	−	++	++	6 (Good)
Norlund et al. [27]	+	+	−+	−	++	++	5 (Good)
Tveito et al. [28]	+	−	++	−	+	−	1 (Insufficient)

− = Minus one, insufficient; −+ = zero, neutral/sufficient; + = one, good; ++ = two, very good. A criteria is also ranked with a −+ in case it was inapplicable to the article or in case it cannot be identified based on the text in the article

Methodological quality score of quantitative studies: −1 to 2 (insufficient), 3–5 (moderate), 6–8 (good), 9–11 (very good). Methodological quality of systematic reviews: −4 to 0 (insufficient), 1–4 (moderate), 5–8 (good), 9–12 (very good)

Methodological quality ranges: quantitative studies from −1 to 11, systematic literature reviews ranges from −4 to 12. Mean scores are calculated when a criteria existed of multiple sub criteria. These mean scores were taken into account in the overall calculation of quality

^a^Final quality scores are calculated by adding up all pluses and subtracting all minuses

In general, the quantitative studies had moderate to good quality, relating to their designs, study populations, control groups and data analyses. Studies [11–13] were of the best quality because of their longitudinal designs, sufficiently large, heterogeneous study populations and adequate control groups. These studies also provided a complete description of the outcome variables, which also matched study aims. To measure the outcomes, instruments were used that are likely to be accepted by the relevant profession. Data were analysed with advanced techniques such as multilevel regression analyses.

The quality of four of the selected systematic literature reviews was good to very good. Review [14] was of the best quality. This review was based on an adequate research question, good search methods, selection, quality evaluation (and description of this procedure), data extraction and description of original studies. It included a meta-analysis and described potential sources of heterogeneity of studies included in the review.

### Intervention Characteristics and Their Effect on RTW

The interventions that were studied as well as their effects on RTW varied largely (Table 5). All interventions were compared to care as usual or a control treatment or to the results of similar studies. In one study, a comparison was made between the number of sickness absence days before and after the intervention in a single group of employees (pre/post test, no control) [15]. This study reduced annual sick leave days for 2 years. We refer to this as a positive effect on RTW.

**Table 5:** Description and effectiveness of the interventions

Study	Target population	Intervention and care as usual	Study outcomes most relevant for this review (operationalisation of return to work/sickness absence)	Effectiveness (on outcomes relevant to this study)
Brouwers et al. [11]	Specific: only employees with specific diagnosis (emotional and stress related complaints)	Activating counselling/control group: care as usual	Sick leave duration (days): period between first day of absence and return to work	No effect on sick leave duration
van der Feldtz-Cornelis et al. [12]	Specific: only employees with specific diagnosis (depressive, anxiety and somatoform disorders)	(1) Training of occupational physicians in diagnosis and treatment (2) supportive psychiatric consultations (3) training of psychiatrist/Control group: care as usual	Time to return to work: period between onset of sickness leave due to mental disorder and full return to work, for at least 4 weeks without relapse	Full RTW at 3 months follow up** survival analysis: return to work occurred 122 (intervention) and 190 days (control) after intervention
Mortelmans et al. [13]	Generic: all employees on sickness absence irrespective of their specific medical diagnosis	Structured and circular information exchange by communication form/control group: occupational physician filled out the communication form and delivered to the researcher.	Return to work rate/median gradual return to work duration in days	No effect on return to work rate. Relative risk: 1.03 (95% CI 0.93–1.13)/no effect on gradual return to work rate. Relative risk: 1.24 (95% CI 0.52–2.97). No difference in median duration of gradual return to work (62 days)
Bogefeldt et al. [18]	Specific: only employees with specific diagnosis (low back pain)	*Group 1*: stay active therapy (e.g. exercise), stretching, manual therapy. *Group 2*: stay active therapy, stretching, manual therapy, corticosteroid injections/control Group 1: stay active therapy Group 2: stay active therapy, stretching	Return to work rate./Sick leave in days (number of days times sick leave extent)	Increase return to work after 10 weeks** (hazard ratio 1.62, 95% CI, 1.006–2.60, *P* < 0.05) and among those on sick leave at baseline, significantly fewer were still on sick leave** (ratio 0.35, 95% CI, 0.13–0.97, *P* < 0.05)/(no effect after 2 years)
Bültmann et al. [20]	Specific: only employees with specific diagnosis (musculoskeletal complaints or low back pain)	Systematic multidisciplinary work disability screening, development and implementation of work rehabilitation plan/control group: care as usual	Cumulative sickness absence hours, time intervals: 0–3, 3–6, 6–12, 0–6, 0–12 months	Lower number of sickness absence hours during intervals 0–6, 6–12 and 0–12**/no effect during intervals 0–3–3–6
Fleten and Johnsen [24]	Specific: only employees with specific diagnosis (musculoskeletal or mental disorders)	General information letter on possible work related measures if sick-listed/control group: care as usual	Length of sick leaves in calendar days	Reduction mean length of sick leaves in subgroups with mental disorders, rheumatic disorders, arthritis and in overall sick leaves lasting 12 weeks or more**
van der Klink et al. [16]	Specific: only employees with specific diagnosis (adjustment disorders)	Graded activity/control group: care as usual by the occupational physician	Return to work rate: percentage return to work (partial or full) at 3 months/duration of sick leave: days lost until full return to work with correction for partial return to work	Increase return to work rate at 3 months*** shorter duration of sick leave** rate ratio: 2.39 (95% CI 1.15–4.95)
Arnetz et al. [19]	Specific: only employees with specific diagnosis (musculoskeletal complaints)	(A) Semistructured interview with employee on social and occupational situation. (B) worksite visits by team for ergonomic assessment and improvements and/or personal vocational training schedule/control group: care as usual	Sick leave: number of sick days at 6 months and at 12 months	Shorter sick leave***/likelihood return to work (odds ratio, OR) at 6 months: 1.9; 95% C.I. 1.0; 3.6, *P* = 0.06/likelihood return to work (OR) at 12 months: 2.5; 1.2; 5.1, *P* < 0.01
Bakker et al. [29]	Specific: only employees with specific diagnosis (emotional and stress related complaints)	Communications by general practitioner to promote functional recovery (e.g. in informing and advising the employee)/control group: care as usual	Sick leave duration (calendar days) from the first day of sick leave until full RTW	No effect on sickness absence duration/hazard ratio: 1.06 (95% CI 0.87–1.29)
Drews et al. [32]	Generic: all employees on sickness absence irrespective of their specific medical diagnosis	Social medicine examination and counselling/control group: care as usual	Duration of sick leave period from first day until at least 315 days/regular employment 1 year after intervention	No effect on sickness absence duration/no effect on likelihood of regular employment at follow up/odds ratio intervention group: 0.76 (95% CI 0.45–1.28)
Hagen et al. [30]	Specific: only employees with specific diagnosis (low back pain)	Physical exercise program, besides control treatment/control group: control treatment	Length of sick leave	No (additional) effect on sick leave
Nystuen and Hagen [31]	Specific: only employees with specific diagnosis (musculoskeletal complaints)	Solution-focused intervention/control group: care as usual	Sick leave: mean length after 12 months/work status (at work or not) 6 months after intervention	No effect on sick leave/no effect on work status
Braathen et al. [25]	Generic: all employees on sickness absence irrespective of their specific medical diagnosis	Multidisciplinary rehabilitation programme/control group: treatment of persons’ own choice	Return to work: percentage of population who resumed work	No effect on return to work
Marhold et al. [23]	Specific: only employees with specific diagnosis (musculoskeletal complaints)	Pain coping skills training, focus on: how to return to work and apply coping skills to occupational risk factors/control group: care as usual	Sick leave (days) over periods of 2 months (2 months before treatment and 6 months follow up)	Patients short-term sick leave (2–6 months): shorter sick leave**/patients long-term sick leave (>12 months): no effect on sick leave
Grossi and Santell [22]	Specific: only employees with specific diagnosis (females on sick leave due to work-related psychological complaints)	Coping with psychological/somatic symptoms of stress/control group: standard individual treatment for stress	Return to work rate: percentage of population who resumed work	No effect on return to work rate
Godges et al. [26]	Specific: only employees with specific diagnosis (low back pain)	Education, counselling on pain management tactics and value of physical activity besides conventional physical therapy/control group: conventional physical therapy	Sick leave duration (days)	Shorter sickness absence duration**
Matheson and Brophy [21]	Specific: only employees with specific diagnosis (low back pain)	Early return to work in transitional light duty work, immediate identification and treatment during work hours/Control group: not applicable	Return to work rate: percentage of population who resumed work/days lost from work	Within 30 days, 94% of all subjects had return to work/increase return to work rate compared to other studies/mean number of days lost from work: 8.8
Weiler et al. [15]	Specific: only employees with specific diagnosis (musculoskeletal complaints)	Outpatient rehabilitation and determination of return to work (regular, assisted or individualised procedure), multidisciplinary team conferences (therapists and Airbus health professionals)/control group: not described	Return to work ratios/annual sick leave days (as compared to before sick leave period)	97% of the Patients returned to their original job at the workplace. Reduction annual sick leave days from 48.8 ± 32.8 days to 34.2 ± 37.3 days***. Intervention stabilised low level annual sick leave days during first 2 years of follow-up
van Oostrom et al. [14]	Specific: only employees with specific diagnosis (musculoskeletal complaints, mental and other health problems)	Interventions directed at work/control group: care as usual or clinical interventions	Time until a lasting return to work: a period of absence from the first day of sick leave to full return to work in previous or equal work for at least 4 weeks without dropping out/Time until first return to work: period of absence from work because of sickness, preceded and followed by period of at least 1 day at work/Cumulative duration of sickness absence: total days of sick leave during follow-up period	Shorter sickness absence duration among workers with musculoskeletal disorders (moderate evidence)/no conclusions on effectiveness in mental health problems and other conditions due to lack of studies/workplace interventions: days until lasting return to work, relative effect hazard ratio 1.70 (CI 95% 1.23–2.35), days until first return to work, relative effect hazard ratio 1.55 (CI 95% 1.32–2.16)/mean cumulative duration of sickness absence: −39.06 days
Carroll et al. [17]	Specific: only employees with specific diagnosis (back pain)	Interventions involving workplace/control group: interventions not involving workplace	Multiple operationalisations of return to work among which time to return to work	Interventions involving employee, health practitioner and employer working together to implement work modifications, were more consistently effective than other workplace-linked interventions
Meijer et al. [7]	Specific: only employees with specific diagnosis (non-specific musculoskeletal complaints)	Several interventions/control group: care as usual or control treatment	Difference in sick leave after treatment as compared to sick leave preceding treatment	Shorter sick leave duration (significance not described): 7 out of 22 treatment programs (inconsistent findings). Essential to effective treatment: knowledge, psychological, physical and work conditioning, possibly supplemented with relaxation exercises
Norlund et al. [27]	Specific: only employees with specific diagnosis (low back pain)	Multidisciplinary interventions/control group: variable	Return to work (measured either directly or indirectly as days of sick leave after start of rehabilitation, with the opportunity to turn sick leave into RTW)	Return to work: difference of effect 21%, relative risk 1.21, 95% CI in favour of the intervention groups (only Scandinavian studies)
Tveito et al. [28]	Specific: only employees with specific diagnosis (low back pain)	Workplace interventions/control group: not described	Lost work days or sick leave due to low back pain	Exercise significantly reduced sick leave duration (limited evidence, level of significance not described)/interventions to treat low back pain have positive effects on sick leave (moderate evidence, levels of significance not described)/no evidence of effect on sick leave from educational intervention, pamphlet, back belts/limited evidence that multidisciplinary interventions have no effect on sick leave (level of significance not described)

** *P* < 0.05. *** *P* < 0.01

Studies are listed in order of methodological quality

Table 6 shows the characteristics of each intervention. The interventions are listed in order of the intervention studies’ methodological quality.

**Table 6:** Intervention characteristics

Study	Timing of intervention, early intervention: starts within 6 weeks of absence	Care professionals involved, multidisciplinary	Planning of activities to support RTW, time contingency, activities followed time schedule	Target population	Character of activities to support RTW, explicit actions to stimulate RTW^a^	Intensity^b^	Employee and employer role, decision authority
Brouwers et al. [11]	No/no restrictiction	No	Yes	Specific diagnosis	Decision RTW: no Gradual exposure: no Work-related adaptations: no	Moderate	Yes
van der Feldtz-Cornelis et al. [12]	No/no restrictiction	Yes	No	Specific diagnosis	Decision RTW: no Gradual exposure: no Work-related adaptations: no	Low or variable	No
Mortelmans et al. [13]	No/no restrictiction	Yes	No	Generic: all absent employees	Decision RTW: no Gradual exposure: no Work-related adaptations: no	Low or variable	No
Bogefeldt et al. [18]	No/no restrictiction	Yes	No	Specific diagnosis	Decision RTW: no Gradual exposure: no Work-related adaptations: no	Low or variable	No
Bültmann et al. [20]	No/no restrictiction	Yes	No	Specific diagnosis	Decision RTW: no Gradual exposure: no Work-related adaptations: yes	High	Yes
Fleten and Johnsen [24]	No/no restrictiction	No	Yes	Specific diagnosis	Decision RTW: no Gradual exposure: no Work-related adaptations: no	Low or variable	Yes
van der Klink et al. [16]	Yes	Yes	Yes	Specific diagnosis	Decision RTW: no Gradual exposure: no Work-related adaptations: no	High/moderate	Yes
Arnetz et al. [19]	No/no restrictiction	Yes	No	Specific diagnosis	Decision RTW: no Gradual exposure: no Work-related adaptations: yes	Low or variable	No
Bakker et al. [29]	No/no restrictiction	No	No	Specific diagnosis	Decision RTW: no Gradual exposure: no Work-related adaptations: no	High	Yes
Drews et al. [32]	No/no restrictiction	No	No	Generic: all absent employees	Decision RTW: no Gradual exposure: no Work-related adaptations: no	Low or variable	Yes
Hagen et al. [30]	No/no restrictiction	Uncertain	No	Specific diagnosis	Decision RTW: no Gradual exposure: no Work-related adaptations: no	High	No
Nystuen and Hagen [31]	No/no restrictiction	No	No	Specific diagnosis	Decision RTW: no Gradual exposure: no Work-related adaptations: no	High	Yes
Braathen et al. [25]	No/no restrictiction	Yes	Yes	Generic: all absent employees	Decision RTW: no Gradual exposure: no Work-related adaptations: no	High	Yes
Marhold et al. [23]	No/no restrictiction	No	Yes	Specific diagnosis	Decision RTW: yes Gradual exposure: no Work-related adaptations: no	High	No
Grossi and Santell [22]	No/no restrictiction	Yes	Yes	Specific diagnosis	Decision RTW: no Gradual exposure: no Work-related adaptations: no	High	Yes
Godges et al. [26]	No/no restrictiction	No	No	Specific diagnosis	Decision RTW: no Gradual exposure: no Work-related adaptations: no	Low or variable	No
Matheson and Brophy [21]	No/no restrictiction	Yes	Yes	Specific diagnosis	Decision RTW: yes Gradual exposure: yes Work-related adaptations: no	Low or variable	No
Weiler et al. [15]	No/no restrictiction	Yes	Yes	Specific diagnosis	Decision RTW: yes Gradual exposure: no Work-related adaptations: yes	High	No
van Oostrom et al. [14]	No/no restrictiction	Yes	No	Specific diagnosis	Decision RTW: yes Gradual exposure: yes Work-related adaptations: yes	Low or variable	No
Carroll et al. [17]	Yes	Yes	No	Specific diagnosis	Decision RTW: yes Gradual exposure: yes Work-related adaptations: no	Low or variable	No
Meijer et al. [7]	No/no restrictiction	Uncertain	No	Specific diagnosis	Decision RTW: no Gradual exposure: no Work-related adaptations: no	Low or variable	No
Norlund et al. [27]	No/no restrictiction	Uncertain	No	Specific diagnosis	Decision RTW: no Gradual exposure: no Work-related adaptations: no	Low or variable	No
Tveito et al. [28]	No/no restrictiction	Uncertain	No	Specific diagnosis	Decision RTW: no Gradual exposure: no Work-related adaptations: no	Low or variable	No

*RTW* return to work

Criteria and sub criteria of a characteristic (for example A, B) are in line with the criteria for evaluating the composition of the included interventions (Table 3)

Studies are listed in order of methodological quality

^a^Three types of actions to stimulate the employee to return to work, which are: making a decision about actual return to work (when and/or how it takes place), gradual exposure to the workplace (for example when employees resume work for a limited but increasing number of hours per week) and implemented work-related adaptations, e.g. workplace (such as ergonomic improvements of furniture)

^b^High: ≥10 h, more than 1 session/moderate: <10 h, more than 1 session/low: once or variable

Based on Tables 5 and 6 we can describe characteristics of interventions that facilitate RTW:

*Timing of intervention: early.* Both interventions that started ‘early’ in the RTW process, namely in employees who were absent for 2 weeks [16] and 2–6 weeks of absence [17] facilitated RTW.

*Care professionals involved:* *multidisciplinary.* Multidisciplinary interventions included care providers and professionals from multiple disciplines such as general practitioners and physiotherapists [18] employer, case managers, occupational therapists/ergonomists [19], occupational physicians (OPs), occupational physiotherapists, chiropractors, psychologists and social workers having the role of case workers maintaining contact with the workplace and municipal case managers [20], OPs and psychiatrists [12].

Multidisciplinary interventions appeared to support RTW in physical complaints [14, 15, 17–21]. Two high quality studies showed that interventions that included contact with the employer/workplace improved RTW at 12 months follow up in employees with musculoskeletal complaints [19, 20]. The majority of the multidisciplinary interventions in psychological complaints were effective as well [12, 16]. However, one study did not show significant effects of multidisciplinary intervention in psychological complaints [22].

*Planning of activities to support RTW: time contingent.* In time contingent interventions, activities took place according to a pre-defined time schedule such as a treatment protocol prescribing the total number of sessions and the topics to be addressed in each session. Overall, evidence regarding the effect of time contingent interventions was inconsistent. Some interventions resulted in an earlier RTW [15, 16, 21, 23, 24], while others showed no significant effect on RTW [11, 22, 25]. Findings differed when subgroups are considered. Time contingent interventions were effective in physical complaints [15, 21, 23, 24]. Evidence was inconsistent about the effectiveness of time contingent interventions in psychological complaints. One intervention was effective [16], while two others showed no positive effects [11, 22].

*Target population: generic or specific.* Evidence regarding the effect of interventions targeted at workers with specific diagnoses such as low back pain or adjustment disorders (specific interventions) was inconsistent. A considerable part of these interventions had a positive effect on RTW [12, 14–21, 23, 24, 26–28]. Other interventions targeted at employees with specific diagnoses had no (significant) effect on RTW [11, 22, 29–31].

Interventions targeted at all absent workers (generic interventions: irrespective of a specific diagnosis) showed no significant effect on RTW [13, 25, 32].

*Character of activities to support RTW: interventions including explicit actions to stimulate the employee to RTW.* Interventions including actions to stimulate the employee to RTW improved RTW outcomes. All these interventions were evaluated only in employees with physical complaints. For example, interventions including decision making on RTW or RTW as part of the intervention all facilitated RTW [15, 17, 21, 23]. Similarly, interventions covering gradual exposure to the workplace, such as progressively augmented work tasks or partial RTW, had a positive effect on RTW [14, 17, 21]. Finally, interventions including the implementation of work related adaptations, e.g. ergonomic improvements of furniture facilitated RTW [14, 15, 19, 20].

*Intensity: high, moderate or low.* Evidence regarding high intensity interventions (>10 h divided over multiple sessions) was inconsistent. Some of them facilitated RTW [15, 20, 23], while others had no significant effect [22, 25, 29–31]. Evidence regarding interventions having a moderate (<10 h divided over multiple sessions) low (once) or variable intensity was also inconsistent (Tables 5 and 6).

*Employee and employer role: decision authority.* In nine studies, the employee and/or employer had decision authority with respect to activities to support medical recovery/RTW and/or actual RTW [11, 16, 20, 22, 24, 25, 29, 31, 32]. For example, the employee had the opportunity to comment on an RTW plan composed by professionals [20]. Often, only the employee and not the employer was given decision authority, for example to decide on (solutions on bottlenecks for) RTW [11, 16, 20, 29, 31, 32]. Evidence regarding the effect of these interventions was inconsistent. Some facilitated RTW [16, 20, 24] while in the majority of the studies no positive effect on RTW was found [11, 22, 25, 29, 31, 32].

## Conclusion

The aim of this study was to detect and identify characteristics of RTW interventions that generally facilitate return to work (i.e. in multiple target populations and across interventions). Generally, we found two intervention characteristics that consistently facilitated RTW. *Early* interventions, that is, interventions initiated in the first 6 weeks of sickness absence, support RTW in multiple target groups. Early interventions appear to be scarce though. *Multidisciplinary* interventions appear effective to support RTW in physical complaints and in the majority of the studies in employees with psychological complaints. Particularly contact with the employer/workplace improves RTW at 12 months follow up in comparison with usual care for subjects with musculoskeletal complaints.

Moreover, we found two intervention characteristics that were effective in all physical complaints groups: time contingent and activating interventions. *Time contingent* interventions are effective in physical complaints. Evidence on effectiveness of this characteristic for psychological complaints is somewhat inconsistent. *Activating* interventions such as gradual RTW are relatively scarce and only found in studies about physical complaints.

Evidence is inconsistent about the effectiveness of interventions targeted at employees with specific diagnoses (although in more than half of the studies with this type of intervention, the results are positive), interventions of varying intensity and interventions covering employee and/or employer decision latitude.

The results of this review show that generic interventions, targeted at all employees on sick leave, irrespective of their diagnoses, show no positive effect.

## Discussion

In this study, we focused on characteristics of RTW interventions that generally were effective. The wide range of target populations and interventions may have diluted the more specific findings though. Therefore, we assessed the effectiveness of the intervention characteristics in physical- and psychological complaints separately. It appeared that early and multidisciplinary interventions were effective in both target groups, while for example time contingent interventions were particularly effective for employees with physical complaints.

Our findings showing the general effectiveness of multidisciplinary intervention suggest the importance of cooperation between care professionals and/or case managers and/or employers to for instance align the medical recovery- and RTW process. Particularly contact with the employer/workplace resulted in improved RTW after 12 months follow up [19, 20]. These interventions may help to find mutually desired work adaptations, supporting the employee’s long-term employability. Second, we found that early intervention stimulates early RTW. Researchers found that early intervention has some other effects than early RTW as well. Early intervention was associated with less repeated sickness absence [33]. At the same time, (early return to) work can be beneficial for the employee’s health [34], hence these effects somehow benefit employees and employers alike.

Additionally, we found that there were more interventions for physical complaints than for psychological complaints. Interestingly, interventions in physical complaints were more often effective than those in psychological complaints. This for example applies to time contingent interventions and might be explained by the following. The course of psychological complaints (such as stress-related disorders) might be more instable than that of physical complaints such as low back pain. Therefore, it might be relatively difficult for people with psychological complaints to follow a pre-defined time schedule for intervention. Also, professionals and employers might be less inclined to do so. Further, it might be that RTW professionals and scientists tend to choose physical complaints as a target population to increase their chances of success. Nevertheless, our study results suggest that early and multidisciplinary interventions are generally effective and should be included in all interventions for RTW.

## Methodological Reflections

This study has some strengths. We performed a comprehensive methodological quality assessment and description of steps that were taken. These features increased the study’s reliability and validity.

Most previous systematic reviews [7, 17, 27, 28] focused on one specific target population such as back pain. We applied our taxonomy of intervention characteristics to multiple target populations including psychological complaints and musculoskeletal complaints. Our study results regarding early and multidisciplinary intervention hold for multiple target populations. This enhanced our current knowledge of strategies to support RTW.

However, our study also has some limitations. It was not possible to perform a meta-analysis due to heterogeneity of the outcome measure (RTW) as defined in the included studies.

Our study results showed the effectiveness of interventions initiated in the first 6 weeks of the RTW process and multidisciplinary interventions. This conclusion is based on only two early interventions that we included in our review. This may be insufficient to consider the study results to be a theoretical framework. However, our results may indicate some successful strategies to support RTW.

Surprisingly, we found that activating interventions (for example those including a decision about RTW) support RTW in employees with physical complaints, while we did not find such interventions for employees with psychological complaints. Possibly, interventions for employees with psychological complaints tend to activate in other ways than measured in this study (that is: deciding about RTW, gradual exposure to the workplace and/or implemented workplace adaptations). For example, interventions may primarily focus on regaining feelings of control and support subjects’ own responsibility to identify and solve bottlenecks for participation [11, 16].

Our taxonomy may not have detail enough to inform professionals in RTW such as OPs about the exact content of appropriate interventions (for example the content of contacts with the employer). To the authors’ knowledge though, this is the first study that assessed the effect of intervention characteristics on RTW in a systematic way, and may as such be a good starting point for RTW professionals.

## Implications for Practice and Research

This review focused on intervention characteristics that facilitate RTW. Our findings have implications for practice and research.

In the first place, the results showed the effectiveness of early interventions and multidisciplinary interventions including contact with the employer. Activating interventions were effective, but only found in physical complaints. Early-, multidisciplinary- and activating interventions should be applied more often, especially in psychological complaints. To start early in the RTW process, general practitioners and OPs need to refer employees and employers to these interventions within the first 6 weeks of the employee’s absence. Interventions should incorporate interdisciplinary cooperation between professionals in health care and contact with the employer. A matrix structure may support this cooperation. It is essential that professionals have enough resources such as time for interdisciplinary contacts. In the Netherlands, the employee and employer have a legal responsibility to cooperate with each other in order to support the employee’s RTW [35]. Researchers and policy makers could study the Dutch situation to find tools for involving the employer in employee RTW. Interventions and other care products should empower both the employee and the employer by incorporating explicit measures to stimulate them to realise RTW.

To know the exact content of successful interventions to support RTW, future studies may focus on detailing our taxonomy of intervention characteristics. Researchers may particularly focus on further detailing the effective intervention characteristics such as appropriate cut-off scores for early intervention and the exact content or intensity of multidisciplinary contact between care providers and employers.

In addition, because we found quite some inconsistent results (e.g. regarding the intensity of the intervention or the involvement of the employer), future research should focus on multifactorial analyses such as meta-analyses. This may help to study which individual or combined intervention characteristics facilitate RTW. Researchers should define RTW precisely and include this single definition as an outcome in any study to increase possibilities for meta-analyses.

In this study, we classified intervention characteristics. Researchers can use our taxonomy to classify the characteristics of RTW interventions in future systematic reviews. This would enable comparison of study results and strengthen the evidence about intervention characteristics that support RTW.

Finally, we found very few early interventions, despite their wide use by professionals (e.g. by many OPs and employers). The gap between research and practice appears to be large. To support evidence-based practice, we advise more cooperation between professionals in practice and research, for example in formulating research questions.
